# Long-term results and recurrence patterns from SCALOP: a phase II randomised trial of gemcitabine- or capecitabine-based chemoradiation for locally advanced pancreatic cancer

**DOI:** 10.1038/bjc.2017.95

**Published:** 2017-04-04

**Authors:** C N Hurt, S Falk, T Crosby, A McDonald, R Ray, G Joseph, J Staffurth, R A Abrams, G Griffiths, T Maughan, S Mukherjee

**Affiliations:** 1 Centre for Trials Research, Cardiff University, 6th Floor, Neuadd Meirionnydd, Heath Park, Cardiff CF14 4YS, UK; 2 Bristol Haematology and Oncology Centre, Bristol BS2 8ED, UK; 3 Velindre Cancer Centre, Velindre Hospital, Velindre Road, Cardiff CF14 2TL, UK; 4 Beatson West of Scotland Cancer Centre, 1053 Great Western Road, Glasgow G12 0YN, UK; 5 Department of Radiation Oncology, Rush University Medical Center, 500 S. Paulina, 013 Atrium Building, Chicago, IL 60612, USA; 6 Southampton Clinical Trials Unit, University of Southampton, Southampton General Hospital, Tremona Road, Southampton SO16 6YD, UK; 7 CRUK MRC Oxford Institute for Radiation Oncology Gray Laboratories, Oxford University, Oxford OX3 7DQ, UK

**Keywords:** pancreatic, chemoradiotherapy, phase II, randomised, trial, gemcitabine, capecitabine

## Abstract

**Background:**

SCALOP, a randomised, phase II trial, tested the activity and safety of gemcitabine (GEM)-based and capecitabine (CAP)-based chemoradiation (CRT) for locally advanced pancreatic cancer (LAPC). Here we present the long-term outcomes.

**Methods:**

Eligibility: histologically proven LAPC ⩽7 cm. Following 12 weeks of induction GEMCAP chemotherapy (three cycles: GEM 1000 mg m^−2^ days 1, 8, 15; CAP 830 mg m^−2^ days 1–21 q28 days) patients with stable/responding disease, tumour ⩽6 cm, and WHO Performance Status 0–1 were randomised to receive one cycle GEMCAP followed by CAP (830 mg m^−2^ b.d. on weekdays only) or GEM (300 mg m^−2^ weekly) with radiation (50.4 Gy per 28 fractions).

**Results:**

One-hundred fourteen patients (28 UK centres) were registered between 24 December 2009 and 25 October 2011, and 74 were randomised (CAP-RT=36; GEM-RT=38). At the time of this analysis, 105 of the 114 patients had died and the surviving 9 patients had been followed up for a median of 10.9 months (IQR: 2.9–18.7). Updated median OS was 17.6 months (95% CI: 14.6–22.7) in the CAP-CRT arm and 14.6 months (95% CI: 11.1–16.0) in the GEM-CRT arm (intention-to-treat adjusted hazard ratio (HR): 0.68 (95% CI: 0.38–1.21, *P*=0.185)); median progression-free survival (PFS) was 12.0 months (95% CI: 10.0–15.2) in the CAP-CRT arm and 10.4 months (95% CI: 8.8–12.7) in the GEM-CRT arm (intention-to-treat adjusted HR: 0.60 (95% CI: 0.32–1.14, *P*=0.120)). In baseline multivariable model, age ⩾65 years, better performance status, CA19.9<613 IU l^−1^, and shorter tumour diameter predicted improved OS. CAP-CRT, age ⩾65 years, better performance status, CA19.9 <46 IU ml^−1^ predicted improved OS and PFS in the pre-radiotherapy model. Nine-month PFS was highly predictive of OS.

**Conclusions:**

CAP-CRT remains the superior regimen. SCALOP showed that patients with CA19.9 <46 IU ml^−1^ after induction chemotherapy are more likely to benefit from CRT.

The SCALOP trial (Selective Chemoradiation in Advanced Localised Pancreatic Cancer) was a randomised multicentre, phase II, open-label trial of GEM-CRT *vs* CAP-CRT following four cycles of GEMCAP chemotherapy in LAPC. It was developed to assess the activity, safety and feasibility of delivering high-quality pancreatic CRT across multiple centres in the UK and to determine the relative benefits and toxicity of gemcitabine *vs* capecitabine as concurrent chemotherapy. To date, this is one of the largest trials testing the approach of induction chemotherapy followed by consolidation CRT and the trial results were reported after the primary end-point (9-month progression-free survival (PFS)) was reached (Mukherjee *et al*, 2013). The 9-month PFS was 62.9% (80% CIs: 50.6–73.9%, *n*=35) in the CAP-CRT arm and 51.4% (80% CIs: 39.4–63.4%, *n*=35) in the GEM-CRT arm (median PFS 12.0 *vs* 10.4 months, hazard ratio (HR)=0·60, *P*=0·11) and the OS was reported to be significantly superior in the CAP-CRT arm (median OS 15.2 *vs* 13.4 months, HR=0.39, *P*=0.012). More patients in the GEM-CRT arm experienced grade 3/4 haematological (18.4% *vs* 0%, *P*=0.008) and non-haematological (26.3% *vs* 11.8%, *P*=0.12) toxicities during CRT. The quality of life results from the trial also favoured the CAP-CRT arm (Hurt *et al*, 2015). The RT Quality Assurance results from this trial have also been previously reported (Fokas *et al*, 2015, 2016).

When the initial results were published previously, 46 of the 74 randomised patients had died. At the time of the analysis presented in this manuscript 69 of the 74 randomised patients had died and we have looked at the updated survival outcomes including multivariable analysis of prognostic factors, patterns of failure, and implications for future research.

## Materials and methods

The trial design, treatment options, eligibility criteria, and follow-up modalities were previously reported in detail (Mukherjee *et al*, 2013). In summary, the trial included patients with the following key eligibility criteria: age >18 years; histologically or cytologically proven malignancy of the pancreas that was locally advanced, non-metastatic, and inoperable (or operable but patient medically unfit for surgery); tumour diameter ⩽7 cm; WHO performance status (PS) 0–2; adequate haematological, liver, and renal function. Lymphoma or neuroendocrine tumours of the pancreas were excluded. Following 12 weeks of induction, GEMCAP chemotherapy (3 cycles: GEM 1000 mg m^−2^ days 1, 8, 15; CAP 830 mg m^−2^ days 1–21 q28 days), patients had a re-staging CT scan and those with: responding or stable disease according to Response Evaluation Criteria in Solid Tumours (RECIST) criteria (version 1.1); tumour diameter ⩽6 cm; WHO PS 0–1; haematological, liver, and renal function as for registration; and no greater than 10% weight loss from that at baseline, were randomised (1 : 1 minimisation with a random element (80 : 20) stratified by recruiting hospital, WHO PS (0 or 1) and disease location (head or body/tail)) to receive a further cycle of GEMCAP followed by either CAP (830 mg m^−2^ b.d. on days of RT only) or GEM (300 mg m^−2^ weekly) with radiation (50.4 Gy per 28 fractions with weekends off). Prospective RT quality assurance was mandated (Fokas *et al*, 2016). The primary end-point was 9-month PFS. Secondary end points included OS, local and distant PFS (LPFS and DPFS), objective disease response, toxicity, treatment compliance, and quality of life (EORTC C30 and PAN26). QOL, WHO PS, and CA19.9 were collected at baseline, week 17 (just prior to radiotherapy), week 26, 39, and 52, CT scans and RECIST assessments were mandated at baseline, week 13 (prior to randomisation), week 26, week 39, and week 52. Treatment following progression was as per institutional practice and no specific regimen was recommended. The trial was approved by a UK multicentre ethics committee and individual informed consent was obtained from all participants.

All statistical analyses were pre-planned and conducted using Stata SE 14 (Stata Corporation, College Station, TX, USA). We calculated survival from the date of registration to when an event occurred, that is, progression and death from any cause for PFS, and death from any cause for OS. Patients who were event free were censored at the time they were last known to be event free. We estimated event time distributions with the Kaplan–Meier method and compared OS and PFS with HRs from Cox regression in univariable models and multivariable models. Global health status (GHS) from the EORTC C30 was included in the baseline model as it has been found to be prognostic elsewhere (Quinten *et al*, 2014). We tested the proportional hazards assumption of each model with Cox–Snell residuals and Schoenfeld’s global test. We conducted an updated analysis of OS and PFS using the mature data in the intention-to-treat and per-protocol (patients starting CRT) population both unadjusted and adjusted for the randomisation stratification variables. In addition, we used the above methods to ascertain which variables were prognostic at two separate points in the treatment pathway:

- Which baseline variables were prognostic of OS for all registered including those patients who were not randomised? This analysis used date of registration as the start of the time period.
- Which variables at the start of radiotherapy were prognostic of OS and PFS? This per protocol analysis used date of start of radiotherapy as the start of the time period.

In the multivariable models, we included all variables thought a priori to have a prognostic effect and included recruitment centre as a random frailty effect. In order to obtain cut-offs for CA19.9 that can be validated in future studies, ROC analysis was used with 12-month OS as the end point. We calculated HRs for GHS for every 10-point difference in scores as has been done elsewhere (Quinten *et al*, 2014) We did not adjust for multiplicity in these primarily hypothesis generating analyses.

## Results

### Study population

**Figure 1:**
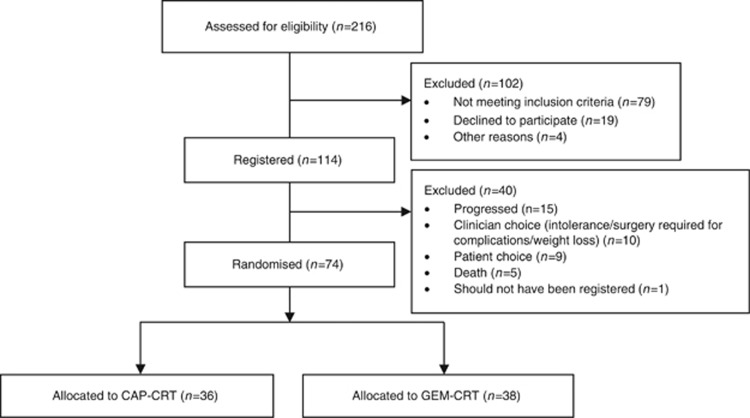
**CONSORT flow diagram of trial participants.**

The study population was described in detail in the first report (Mukherjee *et al*, 2013). Between 24 December 2009 and 25 October 2011, a total of 114 patients were registered into the trial from 28 hospitals across the UK (Figure 1) and following induction chemotherapy, 74 patients were eligible for randomisation (CAP-CRT=36; GEM-CRT=38). Median age was 64.3 (IQR: 57.7–70.3), 55.3% (*n*=63) were male, the mean estimated longest diameter of primary lesion was 4.2 cm (s.d.: 1.5), and WHO PS 0/1/2 was 48.3% (*n*=55), 44.7% (*n*=51), 7.0% (*n*=8), respectively.

### Updated analysis in all registered patients

**Figure 2:**
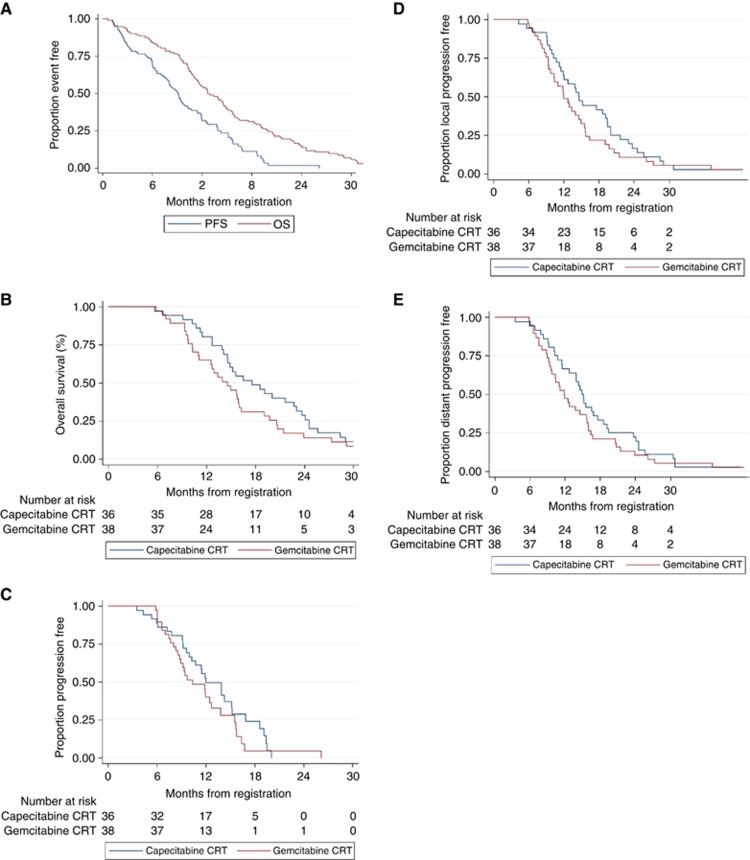
**Kaplan–Meier curves of survival.**(**A**) OS and PFS (all registered patients). Median OS: 12.6 months (95% CI: 11.3–14.9). Median PFS: 9.2 months (95% CI: 7.7–10.3). (**B**) OS by treatment arm (randomised patients). *Median OS:* CAP-CRT: 17.6 months (95% CI: 14.6–22.7). GEM-CRT: 14.6 months (95% CI: 11.1–16.0). *HR:* unadjusted: 0.73 (95% CI: 0.46–1.18, *P*=0.203). Adjusted: 0.68 (95% CI: 0.38–1.21, *P*=0.185). (**C**) PFS by treatment arm (randomised patients). *Median PFS:* CAP-CRT: 12.0 months (95% CI: 10.0–15.2). GEM-CRT: 10.4 months (95% CI: 8.8–12.7). *HR:* unadjusted: 0.73 (95% CI: 0.44–1.23, *P*=0.244). Adjusted: 0.60 (95% CI: 0.32–1.14, *P*=0.120). (**D**) LPFS by treatment arm (randomised patients). (**E**) DPFS by treatment arm (randomised patients).

This population consisted of the 114 registered patients. At the time of analysis, 105 of the 114 patients had died and the surviving 9 patients had been followed up for a median of 10.9 months (IQR: 2.9–18.7). Figure 2A shows the updated OS and PFS of this population.

### Updated analysis in randomised patients

The intention to treat analysis included the 74 randomised patients. At the time of analysis, 34 out of 36 patients in the CAP-CRT arm and 35 out of 38 in the GEM-CRT arm had died. The surviving five patients had been followed up for a median of 12.2 months (IQR: 7.5–41.9). Figure 2B and C shows the updated OS and PFS. Median OS was 17.6 months (95% CI: 14.6–22.7) in the CAP-CRT arm and 14.6 months (95% CI: 11.1–16.0) in the GEM-CRT arm (HR: adjusted: 0.68 (95% CI: 0.38–1.21, *P*=0.185); unadjusted: 0.73 (95% CI: 0.46–1.18, *P*=0.203)). Median PFS was 12.0 months (95% CI: 10.0–15.2) in the CAP-CRT arm and 10.4 months (95% CI: 8.8–12.7) in the GEM-CRT arm (HR: adjusted: 0.60 (95% CI: 0.32–1.14, *P*=0.120); unadjusted: 0.73 (95% CI: 0.44–1.23, *P*=0.244)). Although both end points still favoured CAP-CRT, neither achieved statistical significance in either unadjusted or adjusted models.

In a per protocol analysis that excluded the two randomised patients who did not start chemo-radiotherapy (two patients in the CAP-CRT due to disease progression), the adjusted and unadjusted HRs for OS were 0.61 (95% CIs: 0.34–1.10, *P*=0.101) and 0.69 (95% CIs: 0.43–1.12, *P*=0.138), respectively and for PFS were 0.50 (95% CIs: 0.26–0.98, *P*=0.043) and 0.67 (95% CIs: 0.40–1.14, *P*=0.143).

The primary end point, 9 months PFS, was highly predictive of OS. The median OS in those who had progressed at 9 months was 11.3 (95% CI: 9.5–14.1) months compared to 20.7 (95% CI: 17.6–24.6) months in those who had not progressed at that time point (HR: 5.2, 95% CI: 1.5–5.6, *P*<0.001).

### Baseline model

Upon ROC analysis, the optimum cut off for baseline CA19.9 for predicting 12-month OS in the 101 registered patients where both CA19.9 data and 12-month OS status were available was found to be ⩾613 IU ml^−1^ (75% correctly classified for 12-month OS, sensitivity=69.8%, specificity=79.6%) (Supplementary Figure 1a).

Of the 114 patients registered into the trial, 8 had CA19.9 missing at baseline and 3 had estimated longest diameter of primary lesion missing. A further 9 patients had EORTC C30 GHS missing leaving 94 patients with full data for analysis of all the baseline prognostic factors. Eighty seven of these patients had died at the time of analysis. The surviving seven patients had been followed up for a median of 10.9 months (IQR: 2.8–18.7) after registration into the trial. Age ⩾65 years, better performance status, lower CA19.9 (<613 IU l^−1^), and shorter tumour diameter (analysed as continuous variable) were significantly predictive of improved OS in multivariable analysis (Table 1). The same variables were significant in sensitivity analysis that excluded the EORTC C30 GHS variable (*n*=103). In a further sensitivity analysis that also excluded baseline CA19.9 (*n*=111), age becomes non-significant.

### Start of radiotherapy model—OS

Upon ROC analysis, the optimum cut-off for pre-radiotherapy (week 17) CA19.9 for predicting 12-month OS in the 56 randomised patients who started radiotherapy and where both CA19.9 data and OS status at 12-month were known was found to be ⩾46 IU ml^−1^ (71% correctly classified for 12-month OS, sensitivity=61.5%, specificity=64.3% Supplementary Figure 1b). Proportional change in CA19.9 between baseline and week 17 was less able to predict 12-month OS (area under ROC curve=0.6505, graph not shown).

Of the 74 patients randomised in SCALOP, 2 patients progressed prior to the start of radiotherapy leaving 72 patients in the per protocol analysis. CA19.9 at week 17 was missing in 14 patients, tumour volume measurement was missing in 2 patients, and WHO performance status at week 17 was missing in 2 patients, leaving 54 patients with full data in the analysis. CAP-CRT, age ⩾65 years, better performance status (0 *vs* 1–2), and lower CA19.9 (<46 IU ml^−1^) were all found to be significantly predictive of improved OS in multivariable analysis (Table 2). In a sensitivity analysis, the multivariable analysis was repeated without CA19.9 (which meant that 67 patients could be used); CAP-CRT (*P*=0.011), age ⩾65 (*P*=0.030), and better performance status (0 *vs* 1–2) (*P*=0.008) were all still significantly associated with improved OS.

### Start of radiotherapy model—PFS

CAP-CRT, age ⩾65 years, better performance status (0 *vs* 1–3), and lower CA19.9 (<46 IU ml^−1^) were all found to be significantly predictive of improved PFS in multivariable analysis (Table 2). In a sensitivity analysis, the multivariable analysis was repeated without CA19.9 (as above, *n*=67); CAP-CRT (*P*=0.005), age ⩾65 (*P*=0.021), and better performance status (*P*=0.046) were still significantly associated with improved PFS.

LPFS was not superior in the CAP-CRT arm in the multivariable model (*n*=54; HR=0.58; 95% CIs: 0.25–1.36; *P*=0.207) but was found to be superior in a sensitivity analysis that excluded CA19.9 (*n*=67; HR=0.48; 95% CIs: 0.25–0.93; *P*=0.030; Figure 2D).

DPFS was superior in the CAP-CRT arm, both in a multivariable model (*n*=54; HR=0.24; 95% CIs: 0.10–0.58; *P*=0.001) and a sensitivity analysis that excluded CA19.9 (*n*=67; HR=0.36; 95% CIs: 0.19–0.68; *P*=0.002; Figure 2E).

Patterns of progression in the 72 patients who started radiotherapy are shown in Table 3. We performed a *post hoc* analysis looking at the OS of those patients who progressed locally (*n*=14, median OS 12.7 months, 95% CIs: 10.8–20.1) compared to those patients who had metastatic progression first (*n*=22, median OS 10.3 months, 95% CIs: 7.4–15.2). We found that OS was significantly worse in the metastatic group (HR=2.7, 95% CIs: 1.19–6.14, *P*=0.018) in a Cox regression that included the other covariates found to be significant in the model above (age, trial arm, performance status).

## Discussion

The updated intention to treat analysis shows that the median OS of both arms of the trial are better than initially reported, but superior OS and PFS in the CAP-CRT arm is no longer statistically significant. However, adjusted per protocol analysis excluding patients who did not start CRT (due to progression) did show superior PFS in the CAP-CRT arm. In addition, multivariable per protocol analyses suggest that for those patients who started radiotherapy, CAP-CRT is still associated with statistically superior OS, PFS, and DPFS. Age ⩾65 years, better performance status (0 *vs* 1–3) and lower CA19.9 levels, both at baseline and at start of CRT were also predictive of improved outcomes; additionally, maximum tumour dimension was found to be predictive at baseline. Nine-month PFS was found to be highly predictive of OS. Following CRT, patients who progressed with metastatic disease as first site of progression had significantly worse survival than those who presented with local progression.

Since the initial publication of SCALOP, the LAP07 study has been published (Hammel *et al*, 2016). This trial had a 2 × 2 randomisation, included 449 patients and compared (1) chemotherapy alone to chemotherapy followed by consolidation CRT and (2) addition of erlotinib to standard gemcitabine induction chemotherapy. The CAP-CRT used in the trial was similar to the CAP-CRT arm of SCALOP. LAP07 showed inferior survival in the erlotinib arm, and no benefit of adding CRT to single-modality chemotherapy in terms of OS (15.2 *vs* 16.5 months, *P*=0.83). However local progression was significantly lower in the CRT arm (46% *vs* 32%, *P*=0.03) and treatment-free survival favoured CRT (6.1 *vs* 3.7 months, *P*=0.02). In addition, PFS approached statistical significance in favour of CRT (8.4 *vs* 9.9 months, *P*=0.06). The median OS and the low rates of CRT-associated toxicity seen in SCALOP mirror the findings from the LAP07 study providing external validation to likely survival outcomes from induction chemotherapy followed by CAP-CRT. The survival in SCALOP is comparable to outcomes in borderline-resectable pancreatic cancer treated with neo-adjuvant therapy and surgery (Assifi *et al*, 2011).

While debate continues over the role of CRT in LAPC, a CA19.9 cut-off of 46 IU ml^−1^ showed modest specificity and sensitivity of predicting patients who are likely to be alive at 12 months. Although this cut-off requires external validation, such CA19.9 thresholds may help clinicians to decide whether or not to offer CRT in any given patient who has completed induction chemotherapy.

Both local failures (overall 33.3%) and metastatic failures (44.5%) remain high, suggesting the need to optimise both systemic and local components of the treatment and/or the need for further (molecular) selection to confine the use of loco-regional therapy to a smaller group of patients who are more likely to have confined disease. The SCALOP2 trial aims to build on SCALOP and will test the role of intensification of the CRT regimen in patients treated with an effective induction chemotherapy regimen. Nelfinavir is a protease inhibitor, which in pre-clinical studies has been shown to reduce hypoxia and improve vascularity. A small phase II study has shown promising outcome from integrating nelfinavir in a pancreatic CRT schedule (Wilson *et al*, 2016). SCALOP 2 is a multi-centre trial in which 260 patients with LAPC will be registered to receive gemcitabine and nab-paclitaxel induction chemotherapy—patients with stable/responding disease will be randomised to one of five arms: continuing gemcitabine-nab-paclitaxel chemotherapy; conventional CRT (capecitabine with radiotherapy 50.4 Gy per 28 fractions); high-dose CRT (capecitabine with radiotherapy 60 Gy per 30 fractions); conventional CRT with nelfinavir; high-dose CRT with nelfinavir.

While designing the SCALOP trial, we chose the 9-month PFS end point based on the time point that showed the widest separation in survival curves in the original study by Huguet *et al* (2007). We have now shown in a prospective trial that 9-month PFS was highly predictive of OS (HR: 5.2, 95% CI: 1.5–5.6, *P*<0.001), which supports the use of this end point in future phase II trials to allow earlier reporting of results.

It is difficult to explain why age >65years was found to be a favourable prognostic criteria. CRUK statistics (http://www.cancerresearchuk.org/health-professional/cancer-statistics/statistics-by-cancer-type/pancreatic-cancer/survival#heading-One) indicate that increasing age is normally associated with poor survival in pancreatic cancer. The numbers in this study are small and the data need to be interpreted with caution, for example, age becomes non-significant in the baseline model in a sensitivity analysis that excluded variables with missing data. It should also be noted that there is likely to be inherent bias for patient selection in clinical trials and it is therefore possible that older patients who are fit enough to be considered for SCALOP trial were a biologically selected patient group with better prognosis.

A weakness of the study is the amount of missing data in the models. Twenty out of the 114 patients in the baseline model and 18 out of the 72 patients in the start of radiotherapy model were missing covariates. In the former model, this was primarily due to missing EORTC C30 GHS and a number of the smaller centres not being able to analyse for CA19.9; however our sensitivity analyses that excluded these variables reached the same conclusions (although age become non-significant for OS). In the latter model, this was again primarily due to CA19.9; however our sensitivity analyses excluded CA19.9 and reached the same broad conclusions (other than for LPFS).

In summary, this mature analysis shows that radiosensitisation with low-dose gemcitabine remains an inferior regimen, both in terms of efficacy and toxicity, and cannot be recommended in future CRT trials. However, only the per-protocol analysis was significant perhaps reflecting the small size of the study. CA19.9 levels <46 IU ml^−1^ after induction chemotherapy may define a group of patients more likely to benefit from consolidation CRT—however, this requires further validation. Futhermore, 9-month PFS is well correlated with OS and could therefore a useful end point in future phase II pancreatic CRT trials. Future trials need to optimise both systemic and local components of treatment.

## Figures and Tables

**Table 1:** Univariable and multivariable cox regression analysis of OS by baseline characteristics in all patients

	**OS (months)**^a^			**Univariable**			**Multivariable**		
	***n***	**Median**	**95% CIs**	**HR**	**95% CIs**	***P***	**HR**	**95% CIs**	***P***
Age									
<65	52	11.7	9.8–14.6	1.00			1.00		
⩾65	42	14.1	11.1–17.6	0.77	0.50–1.19	0.237	0.54	0.33–0.88	0.013
Sex									
Male	51	13.9	11.3–16.5	1.00			1.00		
Female	43	11.9	9.3–14.1	1.01	0.65–1.56	0.964	1.12	0.69–1.80	0.654
WHO performance status									
0	45	16.5	12.7–21.5	1.00			1.00		
1–2	49	11.1	8.1–12.7	2.05	1.32–3.17	0.001	2.09	1.24–3.52	0.006
CA19.9									
<613	54	16.5	13.9–19.7	1.00			1.00		
⩾613	40	9.7	7.6–10.7	3.38	2.11–5.43	<0.001	4.11	2.38–7.12	<0.001
Global heath status^b^				0.97	0.89–1.06	0.549	0.95	0.85–1.06	0.395
Longest diameter^c^				1.12	0.97–1.29	0.131	1.28	1.08–1.51	0.005

a From registration into SCALOP trial prior to induction CT.

b HRs were calculated for every 10-point difference in scores.

c HRs calculated for every 1 cm increase.

**Table 2:** Univariable and multivariable Cox regression analysis of OS and PFS by characteristics at the start of radiotherapy

	**OS (months)**^a^			**Univariable**			**Multivariable**		
	***n***	**Median**	**95% CIs**	**HR**	**95% CIs**	***P***	**HR**	**95% CIs**	***P***
Trial arm									
GEM	29	9.5	7.3–12.4	1.00			1.00		
CAP	25	13.9	10.1–19.1	0.67	0.38–1.19	0.172	0.40	0.17–0.91	0.029
Age at start of radiotherapy									
<65	24	10.8	6.4–17.1	1.00			1.00		
⩾65	30	12.2	8.3–15.2	0.75	0.41–1.34	0.333	0.20	0.06–0.67	0.009
Tumour site									
Head	46	10.8	8.3–13.9	1.00			1.00		
Body/tail	8	12.5	9.1–26.8	0.63	0.28–1.42	0.264	0.83	0.79–3.71	0.812
Sex									
Male	32	12.2	9.0–15.7	1.00			1.00		
Female	22	10.3	7.3–13.6	1.03	0.58–1.84	0.910	0.51	0.19–1.37	0.183
WHO performance status at week 17									
0	18	12.2	9.0–17.7	1.00			1.00		
1–3^b^	36	10.3	7.7–14.6	1.04	0.58–1.89	0.885	3.94	1.54–10.12	0.004
CA19.9 at week 17									
<46	26	15.2	11.5–19.9	1.00			1.00		
⩾46	28	9.0	6.4–10.8	3.58	1.89–6.78	0.000	5.66	1.72–18.70	0.004
Gross tumour volume^c^				1.00	0.99–1.01	0.672	0.99	0.97–1.01	0.233

	**PFS (months)**^a^			**Univariable**			**Multivariable**		
	***n***	**Median**	**95% CIs**	**HR**	**95% CIs**	***P***	**HR**	**95% CIs**	***P***
Trial arm									
GEM	29	6.0	4.5–8.6	1.00			1.00		
CAP	25	8.3	6.5–11.5	0.69	0.38–1.26	0.223	0.33	0.12–0.89	0.028
Age at start of radiotherapy									
<65	24	5.6	4.1–8.3	1.00			1.00		
⩾65	30	8.2	6.0–9.8	0.79	0.44–1.44	0.450	0.17	0.05–0.58	0.005
Tumour site									
Head	46	7.0	5.2–9.8	1.00			1.00		
Body/tail	8	8.3	4.1–∞	0.79	0.33–1.89	0.598	0.69	0.14–3.42	0.645
Sex									
Male	32	8.3	5.6–10.1	1.00			1.00		
Female	22	6.4	5.0–7.7	1.84	0.97–3.48	0.062	1.87	0.61–5.67	0.271
WHO performance status									
0	18	8.2	4.5–12.9	1.00			1.00		
1–3^b^	36	7.6	5.3–9.8	1.10	0.57–2.12	0.777	3.04	1.10–8.35	0.031
CA19.9									
<46	26	10.1	8.6–15.5	1.00			1.00		
⩾46	28	5.2	4.4–9.7	4.62	2.29–9.29	0.000	9.31	2.25–38.5	0.002
Gross tumour volume^c^				1.00	0.99–1.01	0.560	0.99	0.97–1.01	0.414

a From first fraction of radiotherapy.

b One patient was PS2 and one was PS3—these two patients deteriorated between randomisation at week 12 and at the start of radiotherapy at week 17.

c As calculated by the investigator—HRs calculated for every 1 cc increase.

**Table 3:** Patterns of disease progression at 12 months

	**CAP-CRT (*****N*****=34)**		**GEM-CRT (*****N*****=38)**		**All randomised (*****N*****=72)**	
	***n***	**%**	***n***	**%**	***n***	**%**
Alive and without progression	1	2.9	1	2.6	2	2.8
Died before progression detected^a^	11	32.4	13	34.2	24	33.3
Progressed	22	64.7	23	60.5	46	63.9
Local	8	23.5	6	15.8	14	19.4
Metastatic	11	32.4	11	29.0	22	30.6
Both	3	8.8	7	18.4	10	13.9

a All these patients died with pancreatic cancer as the primary or secondary cause.
