# Supplementary material for: Long-term results and recurrence patterns from SCALOP: a phase II randomised trial of gemcitabine- or capecitabine-based chemoradiation for locally advanced pancreatic cancer
Source: Br J Cancer. 2017 Apr 4;116(10):1264–70. doi: 10.1038/bjc.2017.95 (PMC5482737; doi:10.1038/bjc.2017.95)
Supplement: Supplementary Figure 1 [file bjc201795x1.docx]

Supplementary Figure 1. ROC analysis showing sensitivity and specificity of different cut points for CA19.9 in predicting survival at 12 months

| 1. Baseline CA19.9 in all patients (n=101)   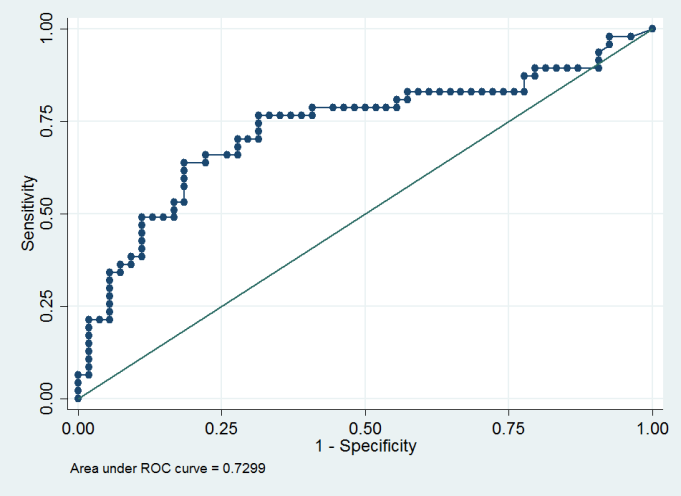 | 1. Pre-radiotherapy CA19.9 (week 17) (n=56)   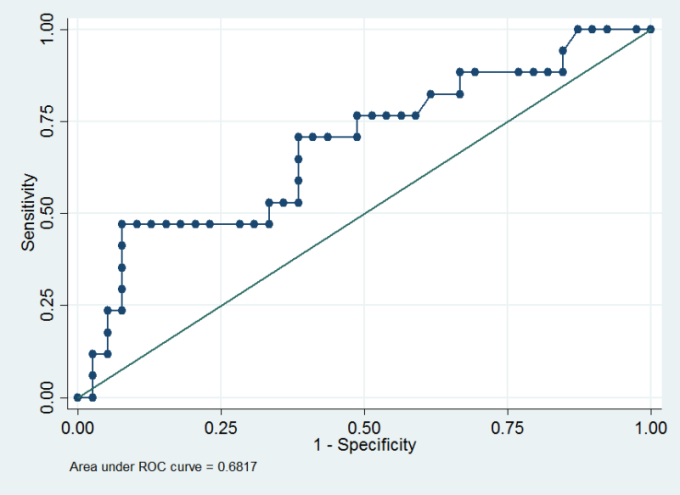 |
| --- | --- |
